# Comprehensive study on the technical aspects of sampling, transporting and measuring radon-in-water

**DOI:** 10.1016/j.jenvrad.2018.11.012

**Published:** 2019-02

**Authors:** Viktor Jobbágy, Heiko Stroh, Gerd Marissens, Mikael Hult

**Affiliations:** European Commission, Joint Research Centre (JRC-Geel), Retieseweg 111, B-2440, Geel, Belgium

**Keywords:** Radon measurements, Drinking water, Proficiency test, Liquid scintillation counting, Gamma-ray spectrometry, Water sampling

## Abstract

The European Commission's Joint Research Centre organizes proficiency tests (PT) on radon-in-water measurements. In order to optimize sampling, transport and measurement methods many tests and small scale proficiency tests have been performed. The waters from natural springs, wells were sampled on-site in glass bottles then transported cooled to the JRC and collaborating laboratories. For the material characterization standard measurement methods based on gamma-ray spectrometry, emanometry and liquid scintillation counting were used. The influence of sampling, transport and sample handling on radon-loss was tested and quantified. It was observed that parameters like container material, filling height, storage temperature and handling can lead to substantial measurement bias due to radon-loss. This high risk for radon-loss from samples can potentially be a general radioprotection problem as doses to the public may be underestimated. Regular air and road transport can be considered adequate means of transport as they have little influence on radon-loss if a suitable glass sample container with flexible cap is used and that it is completely filled. On the basis of this work, modifications to the related standard as best practices are also proposed.

## Highlights

• Biasing parameters and conditions in the radon-in-water analytical chain are detailed.
• The influence of sampling, transport and sample handling on radon-loss was quantified.
• Parameters were optimized to assure the quality of radon-in-water proficiency test.
• Modifications to the related standard as best practices are also proposed.

## 1 Introduction

The JRC-Geel organizes proficiency tests (PTs) of radiological analyses of drinking water and other matrices on the request of the European Commission's Directorate-General for Energy (DG ENER). This is in support of the Euratom Treaty Article 35 and the aim is to check comparability of these measurement results and verification of data submitted to the EC by EU Member States. These PTs are usually linked to a European legal document or directive related to radioactivity in environmental matrices or food/feed. One of the benchmark EU directives in this field is the EURATOM Drinking Water Directive, referred as E-DWD, which was published in 2013 (EURATOM, 2013). This E-DWD covers several naturally occurring radionuclides including ^222^Rn (radon) but excludes thoron (^220^Rn) and actinon (^219^Rn), consequently also does this article. Radon-in-water analysis is one of the most frequently used radiological monitoring methods. Despite this fact, very few PTs have been organized in Europe, largely due to practical problems in implementing such PTs or intercomparisons. Furthermore, the proof of metrological traceability, material homogeneity and stability studies have been missing or incomplete in past PTs.

There are some pitfalls in handling and measuring radon-in-water. Following ISO 5667 standard (ISO 5667-3:2012), when samples are prepared for proficiency tests, stricter requirements have to be set on transport and sampling conditions than for normal routine measurements where analysis is generally done within a short time of a few hours or maximum a day. Radon enters into waters as a result of natural processes like decay of its parent nuclide ^226^Ra but predominantly from dissolution of the surrounding geological environment (rocks, soils) (Moreno et al., 2014; Fonollosa et al., 2016). If a PT provider would like to use natural waters, homogeneity within the individual PT samples has to be assured and proved. In addition, the water will need to contain relatively elevated levels of radon-concentration due to the decay before a participant can measure it since ^222^Rn half-life is only 3.8232 (8) days (Bé et al., 2008). When addressing homogeneity issues one must be aware of phenomena linked to natural variations of ^222^Rn activity concentration (Bourgoignie et al., 1982; Freyer et al., 1997). These characteristics must be tested before the proficiency test by the PT organizer. Once a suitable water source is identified, manipulations linked to sampling, storage and transport that are specific for that source must be designed. The importance of sampling and transport conditions are mentioned in technical documents (ISO 13164-3:2013, ISO 13164-4:2015) but very few numerical values have been published on their contribution to uncertainty and radon-loss (Gruber et al., 2009).

The very first and probably the most important manipulation when a representative sample needs to be taken is sampling at the location of the water source. During this procedure a considerable fraction of radon can be lost. Besides sampling, storage is another important phase in radon analysis. Sometimes, there is a significant time-delay before a sample can be measured due to lack of capacity or other priorities in a lab. Therefore, storage conditions, storage time and container material strongly influence the measurement results. In the past decade many different materials, mainly plastics but glass as well, were studied for radon tightness (Leaney and Herczeg, 2006; Lucchetti et al., 2016; Pujol and Pérez-Zabaleta, 2017; Vesterbacka et al., 2010).

Another parameter to consider and control is temperature. According to the ISO standards, low temperature, preferably lower than the temperature at the time of sampling but above 0 °C to avoid freezing, helps preserving radon in the sample. When transport is needed then the water temperature should be kept as stable as possible because it can influence the outcome of the measurements by affecting the measurement geometry in case of direct analysis due to changes in density and eventually volume of the sample. For indirect measurements that require sub-sampling, the bottle has to be opened whereby the water gets into contact with ambient air so that radon can escape. If the water density increases due to cooling, then the water volume decreases, which results in bubble (headspace) formation in the sampling container where radon can be released from the water and accumulated. The level of degassing and eventually radon-loss during transport strongly depends on the headspace volume, storage temperature and physical agitations like vibrations, shaking movement or accidental package drop.

There are different sampling strategies for samples depending on water sources, e.g. running water or stagnant water as described in the ISO standards (ISO 5667, 2006; ISO 5667, 2012; ISO 13164, 2013). There are some common important considerations in the aforementioned standards. The first one to adjust the water flow if possible to avoid turbulence and air bubbles in the sampling container. Another important consideration is that the sampling container should be completely filled and no air bubbles should be present after closing the container air-tight with the container cap. In a sampling container with head-space, ^222^Rn gas occupies the empty space which changes the counting geometry for ^222^Rn and its progenies and eventually the counting efficiency (^222^Rn should stay in the sample matrix). The ISO standards stress that the importance of avoiding contact between the water and the air during sampling. If sample is taken with a syringe then it has to be done with a gas-tight syringe.

There are international standards dedicated to water radon measurements like ISO 13164-3:2013, ISO 13164-4:2015 and ASTM D5072 – 09 (2016). Besides the technical part, they describe the principles of the methods, some of the sampling issues, transportation and storage conditions. In principle there are three different water radon measurement approaches. The first one uses gamma-ray spectrometry (referred as GS), the second emanometry and the third approach is based on liquid scintillation counting (LSC).

The measurement techniques could be grouped depending on some key aspects, for example detection systems and from radon loss point of view more relevant, application of phase transfer. The easiest technique is by direct measurement using GS not involving any phase transfer and therefore minimizing risk of radon-loss. When phase transfer is involved, ^222^Rn is transferred from the aqueous phase to another phase. In case of, emanometry radon is transferred from liquid to gas phase. When liquid scintillation counting is to be used, the target destination matrix is an organic phase. One can also choose to adsorb radon on solid porous surfaces, like activated charcoal, which enables radon to be measured using GS. More details on the different standard measurement methods are discussed elsewhere (Jobbágy et al., 2017).

Due to the short ^222^Rn half-life, decay correction must be considered. Therefore, it is important to know the sampling time accurately preferably referred to the time standard, Coordinated Universal Time (UTC). Furthermore, correction for decay during measurement is necessary for long measurement times. For example during a 24-h measurement, the ^222^Rn activity decreases by 17% and for a “week-end measurement” of 2.5 days it decays by 36%. Samples with low radon activity concentration should be measured shortly after sample collection (e.g. within one ^222^Rn half-life).

Because very few concrete values have been published on the contribution of sampling, transport and sample handling to uncertainty and radon-loss (Gruber et al., 2009), one of the main scopes of the study was to test and quantify the influence of sampling, transport and sample handling on radon-loss. Special attention was paid to parameters like container material, filling height, storage temperature and handling. We also aimed to establish the optimal transport-storage conditions to secure radon tightness and minimize losses through the whole transport chain to safeguard the high quality of the full scale European PT to be carried out in the autumn 2018. Some of the results are from two so-called Pilot-PTs carried out in 2018 (evaluation PT reports will be published separately).

## 2 Materials and methods

For the purpose of testing radon measurement methods and radon loss, the natural spring waters for the Pilot-PTs were collected from one location in Austria and one in Germany (in total 10 units of 1 L samples from each location). In addition, natural spring and well waters were also collected from several other locations in Belgium and Hungary (3 units of 1 L each location). There was also an in-house prepared radon rich water sample that was produced by using an external emanation source with known ^226^Ra activity. The volume of this sample was 1 L each time.

At JRC-Geel the ISO standard methods were followed for gamma-ray spectrometry, emanometry and two-phase liquid scintillation counting as introduced in the next paragraphs.

High purity germanium (HPGe) detectors were used for gamma-ray spectrometry. The original bottle was placed directly on the endcap of the HPGe-detector. The measurement chamber is continuously flushed with nitrogen that boils off from the liquid nitrogen Dewar used for the detector. The measurements were performed for one up to several half-lives to check the decay and possible ^226^Ra presence. The Full Energy Peak (FEP) efficiency curve was established by measuring volume sources of a stable gel containing a range of standardized radionuclides common for calibration provided by Eurostandard. The efficiency transfer (from calibration container to glass bottle) and coincidence summing corrections were calculated with the Monte Carlo simulation software EGSnrc (Electron Gamma Shower National Research Council, Canada). For the determination of the ^222^Rn activity concentration the following gamma-ray peaks of the daughter nuclides were used (assuming secular equilibrium between ^222^Rn and its daughters): ^214^Pb (295 keV, 352 keV) and ^214^Bi (609 keV, 1120 keV, 1238 keV, 1764 keV).

For liquid scintillation counting, (LSC) plastic 20 mL low-diffusion vials and UltimaGold-F water non-miscible cocktail were used. Two Quantulus 1220 (PerkinElmer) counters were used. The typical counting time was set to 120 min and the measurement started with a delay of more than 3 h after shaking the sample to allow establishing equilibrium between ^222^Rn and its short lived decay products. Efficiency calibration, which includes extraction and counting efficiency, was established by measuring two ^226^Ra standard solutions from Eurostandard and National Institute of Standards and Technology (NIST) at equilibrium with ^222^Rn. Combined extraction and counting efficiencies were found to be between 430 and 470% including radon daughters and without alpha-beta separation mode. Blank samples containing boiled de-ionized water were prepared for background measurements in the same way as the samples.

Background samples were prepared in two different LSC vial materials (low-diffusion antistatic polyethylene, low-potassium glass) to compare their contribution to the background counts. Both vials were equipped with screw caps including aluminum foil liners to achieve sufficient radon tightness. Background samples were prepared by boiling 200 mL deionized laboratory water for 10 min while stirring with a magnetic stirrer to facilitate removing residual radon. Measurements were performed by using Wallac/Perkin Elmer Quantulus counters in alpha + beta counting mode.

Two emanometry methods were tested. The first one uses an ionization chamber (AlphaGuard, Saphymo Gmbh) and the other one a semiconductor (RAD7, Durridge Company Inc.) counting system. For the AlphaGuard sample preparation, an aliquot of water was carefully transferred from the original sampling container by a 100 mL syringe into a gas-tight glass degassing vessel. After closing the bottle airtight, the bottle was connected to the AlphaGuard's closed loop consisting of a buffer security vessel, a circulation pump with flow regulator and the AlphaGuard radon monitor. Radon was de-gassed from the water sample using the circulation pump and transferred to the ionization chamber. Radon activity concentration was calculated using a formula provided by the manufacturer (Saphymo, 2010).

The water sample was transferred from the original sampling container either into a 40 mL or 250 mL gas tight glass bottle very carefully. After closing the bottle airtight, the bottle was connected to RAD7 in a closed loop. Radon was aerated from the water sample using a built in pump for 5 min and transferred through a desiccator column to the detector where ^222^Rn activity concentration was derived from counting the ^218^Po. The radon activity concentration at the time of the analysis was considered as the mean value given by the instrument. This value includes the counting efficiency calibration of the RAD7, the size of the sample vial and the total volume of the closed air loop and the radon degassing efficiency determined by the manufacturer (Durridge, 2015).

## 3 Results and discussions

### 3.1 Comparison of direct and indirect methods

Tests of different measurement methods in our laboratory on six different natural water samples revealed some differences. Usually, lower values were measured in case of indirect methods compared to activity values obtained by the direct method (GS). This could be linked to sample manipulation, when transferring water from the glass bottle to the measurement or degassing container. However, Pujol and Pérez-Zabaleta (2017) observed the opposite their GS results were below those from LSC and emanometry. Comparing our results from the different measurement techniques the highest bias was from the emanometry (using both the RAD7 and AlphaGuard instruments) as presented in Table 1. Under routine conditions the difference from a natural water sample with elevated radon concentration from Germany was up to 25%. Regarding comparison of emanometry to liquid scintillation counting our observations can support the results obtained by Stojkovic et al. (2015) where emanometry (RAD7) has approximately 30% negative bias. We observed a systematic 10–25% of lower results from emanometry measurements than that of LSC. It was found that during sample transfer with a 100 mL syringe or pouring into the measurement container partial radon degassing can occur.

**Table 1:** ^222^Rn massic activities from measuring a natural water sample from Germany. The combined standard uncertainty (k = 1) is given.

Direct method	Indirect methods		
Gamma-ray spectrometry (n = 11)	Liquid scintillation counting (n = 20)	Emanometry (AlphaGuard) (n = 3)	Emanometry (RAD7) (n = 5)
(2299 ± 58) Bq/kg	(2272 ± 68) Bq/kg	(1902 ± 135) Bq/kg	(1850 ± 190) Bq/kg

n: number of individual samples from the same water source.

It was observed that when additional sample transfer is involved (indirect measurement approach) from 1.2 up to 25% radon can be lost comparing to direct measurement results.

The typical LSC and GS measurement uncertainties are usually below 20% (coverage factor, *k* = 1) but emanometry techniques can also reach this level. Depending on the method used and the required sample preparation the sub-sample volume can vary in a wide range from 10 mL (LSC), 40–500 mL (emanometry) up to 1.2 L (GS).

### 3.2 Liquid scintillation counting (LSC) vial material and detection limits

^222^Rn and its short-lived progenies were measured by selecting either the alpha + beta or the alpha-only mode of the LS spectrometer. If alpha-beta discrimination is used, then the discrimination setting is crucial as it can easily lead to erroneous results. Photo- and chemiluminescence may also occur, but the latter mainly in case of single phase cocktails. Photoluminescence can be avoided by sufficient storage time of a few hours in a dark place before starting the measurement.

One of the most important performance indicators of a method is its detection limit (for definition, see ISO 11929:2010). This can be reduced by choosing alpha/beta discrimination and the vial material. Glass vials have a considerably higher intrinsic background, approximately three times higher than the plastic vial. However, radon diffusion can occur in case of a plastic vial while it is drastically suppressed for glass. Using alpha beta discrimination results in lower achievable detection limit but the time and quantity of materials needed is higher. One has to balance what is sufficient for the measurement purpose.

As seen in Table 2, on one hand using glass vials can cause higher background and eventually higher (MDA) but radon diffusion can be totally excluded. On the other hand, low diffusion plastic vials contribute less to radon background but the probability of radon diffusion to the LSC vial wall is higher especially for a longer storage time (>5 days). The detection limits are almost equal despite the increment in the background counts.

**Table 2:** Comparison of background counts and MDA in LSC using glass and low diffusion polyethylene vial. High energy beta protocol without alpha-beta discrimination was set, channels between 50 and 1024 were considered (120 min data acquisition).

Vial material	Number of replicates	Mean counts	MDA* (Bq/kg)
Low diffusion polyethylene	4	277	0.3
Low potassium glass	3	616	0.5

*Minimum Detectable Activity is derived from the Detection Limit introduced by Currie (1968).

All of the detection systems at JRC-Geel comply with the detection limit requirement derived from the E-DWD. They all can measure radon activity concentration down to 10 Bq/L as calculated for the lowest detection limit in case of 100 Bq/L radon parametric level.

### 3.3 Radon tightness of the container material

**Fig. 1:**
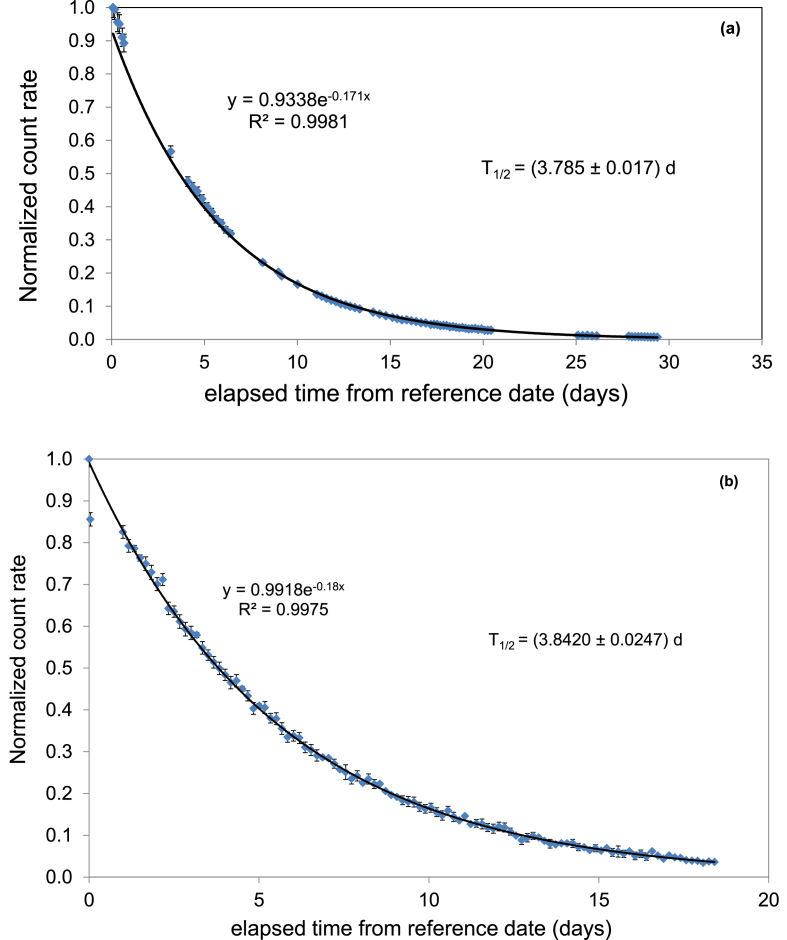
^222^Radon decay curve as a function of time recorded by (a) low-background liquid scintillation counting and (b) high-purity germanium detector.

The tightness of glass containers and low diffusion polyethylene LSC vials was checked with GS and liquid scintillation counting, respectively. An aliquot of a sample with high activity radon was transferred into a sampling bottle or an LSC vial. The glass bottle or polyethylene vial was closed with its cap and placed on the measurement instrument (HPGe detector or LS counter respectively). Measurement data were recorded until radon had decayed so far that only the background was detected (see Fig. 1).

As seen the trend followed the literature radon decay, the deviation from the literature half-life value was less than 0.4% and <1% in case of GS measuring a sample in a glass bottle and a low-diffusion polyethylene LSC vial, respectively. The covered time-spans varied between 18 and 29 days.

In previous studies, polyethylene terephthalate (PET) and polylactic biopolymer (PLBP) were found to be more suitable materials than polyethylene (HD/LDPE) (Leaney and Herczeg, 2006; Lucchetti et al., 2016). After four days of storage in PET/PLBP and PE containers 2% and 15–27% radon-loss was observed, respectively (Lucchetti et al., 2016). In addition to that, Pujol and Pérez-Zabaleta (2017) reported in a recent study that significant radon-loss (2–5%) was observed from polypropylene containers in one day after sampling while radon leakage was negligible from glass and PET vials. They also found that this value increased after seven days to 15–35%. The main factors that might affect radon-loss are surface/volume ratio of the container material, density and wall thickness. Vesterbacka et al. (2010) found that after 5 days, when glass bottles were used, there was much lower radon-loss; below 5%. This value of the radon-loss was consequently included in the uncertainty calculation of this study (as glass bottles were used) when the uncertainty budget of the intercomparison assigned value was established. When radon-loss is expected it should be quantified a correction factor should be provided with an associated uncertainty as well. It has to be stressed that if one wants to be compliant with ISO standards, then glass bottles must be used as sampling containers for radon (ISO 5667-3:2012).

Not only the bottle material but its cap also plays an important role to preserve sample stability and bottle integrity. We observed that bottles with rigid caps tend to break due to temperature changes. We can stress that the cap has to be radon tight and made of flexible material to better resist volume changes due to temperature variations during transport, storage and measurement.

### 3.4 Heat treatment and radon-loss

A sample from a Hungarian spring was subjected to a heat treatment test at JRC-Geel, where it was stored at 45 °C in a climate chamber (Memmert GmbH) for three days. The bottle was also vigorously shaken manually for 5 min to simulate road transport vibration.

**Fig. 2:**
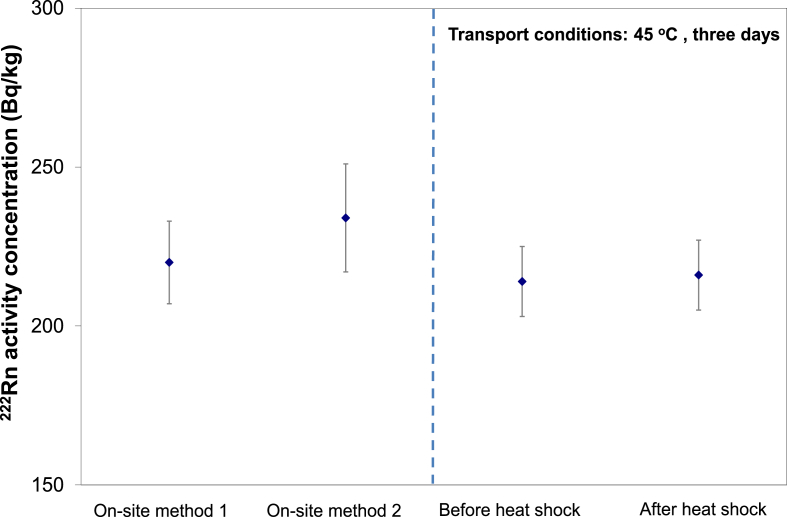
Comparison of radon massic activity results from on-site measurement methods and measurements before and after heat treatment.

The radon massic activity was measured before and after the heat treatment with low-background GS until good counting statistics was reached (<1%). This experiment was repeated two times and results are plotted in Fig. 2.

As seen in Fig. 2, there is no detectable difference between the before and after heat test measurements. The conclusion is that if the bottle is filled without bubbles, tightly capped and if a proper storage container is used then normal air/road transport can deliver samples without significant detectable ^222^Rn loss, even when the samples are exposed to somewhat elevated ambient temperature.

This observation is important in the context of long term transports and warmer climate countries where 45 °C can be reached inside the transport box unless cooling elements are used.

**Fig. 3:**
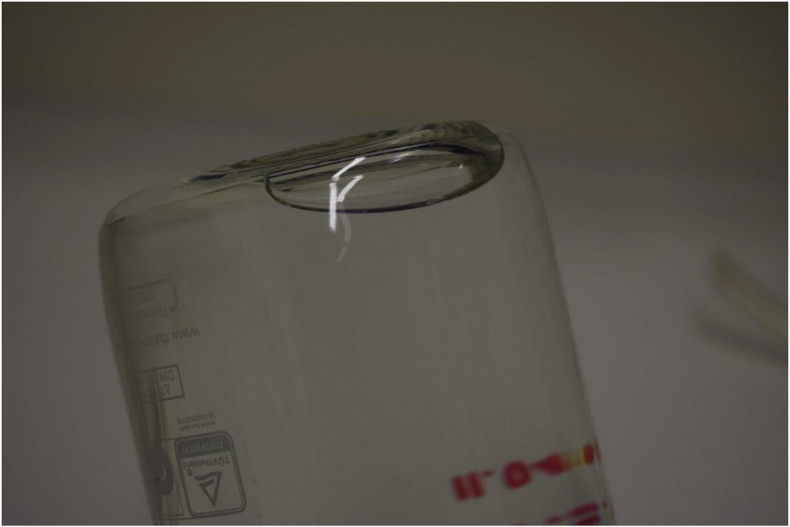
Bubble formation in the sampling bottle after cool storage of sample. Sampling bottle was turned upside down for the better visibility of produced bubble.

Another experiment was performed when samples were warmed up to 45 °C for three days followed by cooling down samples to 3 °C (recommended storage temperature by ISO 5667-3) for three days and then taken out and let the sample be warmed up to ambient temperature 20–21 °C. All these thermal treatment steps were performed in a climate chamber (Memmert GmbH). The first observation was that large bubbles were formed after cooling down samples and these bubbles did not disappear after warming up to ambient temperature. Next, fully filled bottles were placed into a climate chamber cooled down to 3 °C for a day and after taken out let the sample be warmed up to ambient temperature 20–21 °C. The same was observed as after the previous experiment, large bubbles were formed and did not disappear as shown in Fig. 3.

The volume of these bubbles was measured with a graduated volumetric cylinder and the average value was found to be 7.8 mL with the minimum and maximum values being 7.5 and 8.8 mL, respectively.

After opening such a bottle to take a subsample for LSC measurement we obtained up to 8% lower measurement results than when taking a subsample from a fully filled bubble free bottle. Radon can escape from the sample and accumulate in the bubble in the headspace changing the radon distribution in the sampling bottle, especially if physical agitation like vibration accidental shaking happens during transport. This will also affect the GS measurement (changing the geometry) and LSC measurement (within sampling bottle inhomogeneity) results. For this reason, we suggest that drinking water samples should not be cooled down below the water temperature observed during sampling as proposed in the ISO 5667-3 standard on sampling and storage.

A shaking test was conducted by shaking sampling bottles with different headspace for 1 min and results were compared to the unshaken samples from the same bottle as presented in Table 3.

**Table 3:** The effect of shaking samples with different headspace volume on the radon massic activity.

Headspace (bubble) volume in 1 L sampling bottle (mL)	Before shaking	After shaking	^222^Rn loss
8	(50.2 ± 1.1) Bq/kg	(48.0 ± 1.0) Bq/kg	(4 ± 3) %
8	(54.0 ± 1.1) Bq/kg	(51.2 ± 1.1) Bq/kg	(5 ± 3)%
8	(461.5 ± 18.6) Bq/kg	(400.1 ± 16.1) Bq/kg	(15 ± 6)%
390	(655 ± 38) Bq/kg	(150 ± 9) Bq/kg	(77 ± 6)%

If a bottle with already 0.8% of its total volume headspace is exposed to physical agitation like vibration and shaking, radon-loss is enhanced and after opening such a bottle radon-loss can be up to 15%. It is more elevated when the storage bottle is filled up less, leaving more headspace in the bottle (e.g. when approximately 60% of its total volume is filled and exposed to physical agitation radon-loss can reach almost 80% of its original activity).

An additional study would be desired to check the radon-loss at constant headspace as a function of the radon activity of the sample.

### 3.5 Regular air transport test

**Fig. 4:**
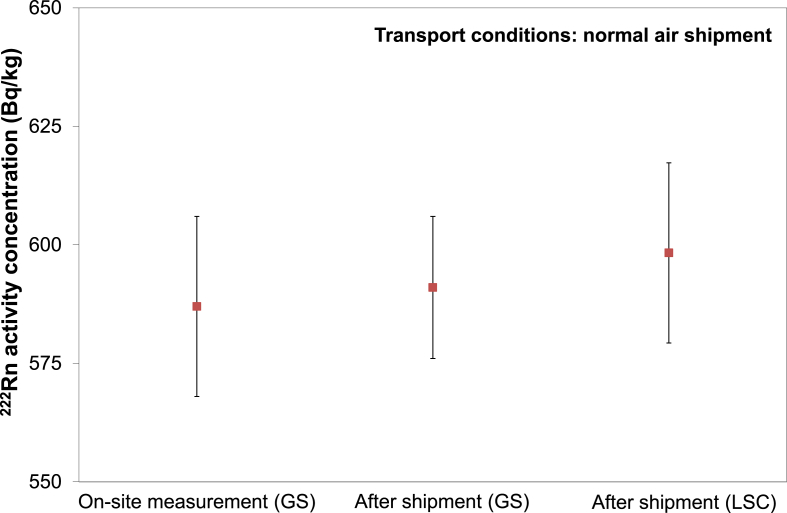
Comparison of radon massic activity concentration before (on-site direct method) and after regular air transport to JRC-Geel.

Air transport was tested when samples were sent from two different locations within Europe (Hungary and Germany) to Belgium. The first sample was transported from Hungary to JRC-Geel (Belgium) in August 2017 during a warm summer period. Then another radon rich water sample was transported from Regensburg to JRC-Geel (Belgium) in October 2017 when the weather was cooler. For both cases, regular cardboard transport boxes filled with plastic cushioning were used. The glass bottles were wrapped in bubble plastic foil and placed in a sealed plastic bag. At this time there was no extra protection against heat exposure during transport (e.g. thermally insulated boxes and cooling elements). The effects of normal air transport were measured by low level GS and LSC measurements where the radon massic activity of samples was measured before and after air transport as presented in Fig. 4.

As can be seen, the measurement results before (on-site measurements) and after regular air transport were in agreement within their standard uncertainties. This suggests that if proper sampling bottle is used, sample stability can be maintained during normal air transport even without extra precaution.

### 3.6 Additional sample transfer for indirect measurements

An indirect measurement is a procedure when extra sample transfer is introduced for some reasons between sampling and measurement container (i.e. sampling bottle is not the same as the container used for the test portion). This is the case usually for liquid scintillation counting and for certain emanometry methods and even for GS when sampling bottle cannot be placed directly on the end cap of the detector. In case of LSC and emanometry a test portion is usually transferred with a syringe with various volumes (most commons are between 10 and 250 mL). The corresponding ISO standard on LSC measurement says gas tight syringe with a needle should be used. However, when we tested this approach we experienced some difficulties during withdrawing a sample with certain types of syringes. One had to pull the piston hard to draw water into the syringe. It was not possible to operate the syringe with one hand. Bubbles were formed which was observed visually even at the most cautious attempt so the test portion was partially degassed. Syringes with different volumes between 10 and 30 mL were tested and a syringe with 30 mL volume was found to be the easiest to manipulate with a wider diameter plastic tube instead of a needle. Therefore, we selected this as our standard syringe. Afterwards, needle substitution options were investigated and a tycoon tube with 5 mm in diameter with a length of 60 mm was found as an optimal solution. This tube is used only for taking the test portion, and then replaced with a needle when ejected under the liquid scintillation cocktail in the vial.

**Fig. 5:**
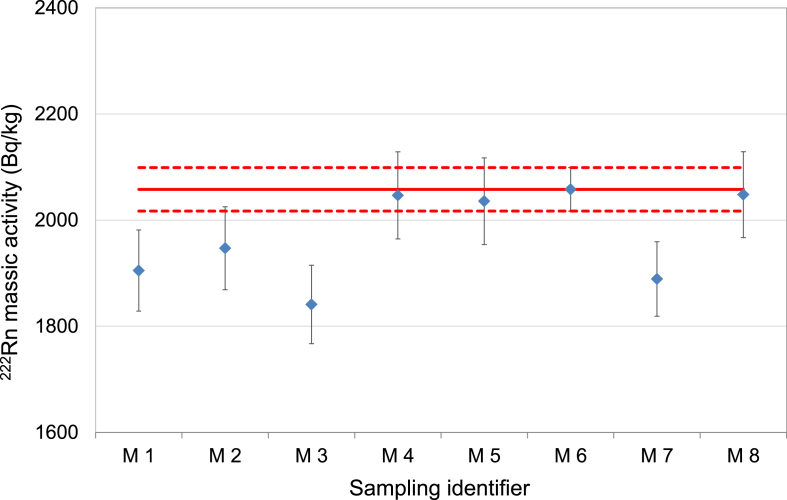
Comparison of different subsampling and transfer approaches to the direct GS measurement (M1: Intense pouring; M2: Careful pouring; M3: Gas tight syringe with needle 1; M4: Gas tight syringe with needle 2; M5: Gas tight syringe with tube and needle; M6: Gama-ray spectrometry; M7: Pipetting with suction; M8: Pipette immersion). The solid red line indicates the ^222^Rn massic activity from the direct measurements (M6) and the dashed red lines represent its combined standard uncertainty. (For interpretation of the references to colour in this figure legend, the reader is referred to the Web version of this article.)

We also tested sub-sampling with a regular 10 mL laboratory pipette to see if it can be in the worst case an alternative to the syringe sampling. Sampling was done first as the pipette tip was fixed to an automatic pipette (suction). Then a 10 mL plastic pipette tip was gently immersed into the sampling bottle until the water level reached 10 mL due to capillary-communicating vessels principle (immersion). Measurement result from the different test portion sampling approaches are compared and presented in Fig. 5.

When comparing the relative differences between the GS measurement result (direct measurement) and the various test portion sampling approaches, it can be observed that one particular gas-tight syringe with needle and tube gave results closer to the GS results than needle sampling. Pipette immersion sampling was found to be suitable with some limitations and possible pitfalls, the difference from this measurement result is within the standard uncertainties and may suggest that it can be an alternative approach to gas-tight syringe with tube-needle combination. However, we also obtained lower measurement results when a pipette with suction was used which is in agreement with the observation of Mazur et al. (2017). This radon-loss is probably due to degassing and controlling the slow motion of analyst's thumb. For this reason we do not suggest this as the first choice of standard sampling method but in case when syringe is not available it can also be an option.

We also made an attempt to quantify radon-loss associated to pouring a water sample from the sampling bottle into a measurement container. There were two different approaches: the first one was pouring along the wall of the measurement container (careful pouring) and the second approach with direct pouring without any precaution, with some bubbles also being formed (intense pouring). Samples for LSC measurements were taken and the results were compared. It can be observed that lower values were obtained after introducing an extra sample transfer but we could not conclude that there is a difference in the mean values between gentle pouring or pouring without any precaution. We also have to note that the pouring approach differs from person to person (more experiments are needed). Measurement results were lower but still within the relative standard uncertainties comparing to the normal sampling procedure. We propose that as part of the best practice extra transfer should be avoided if possible. The organization of a PT involving the participants travelling to the sampling site and doing on-site sampling would be highly valuable.

**Fig. 6:**
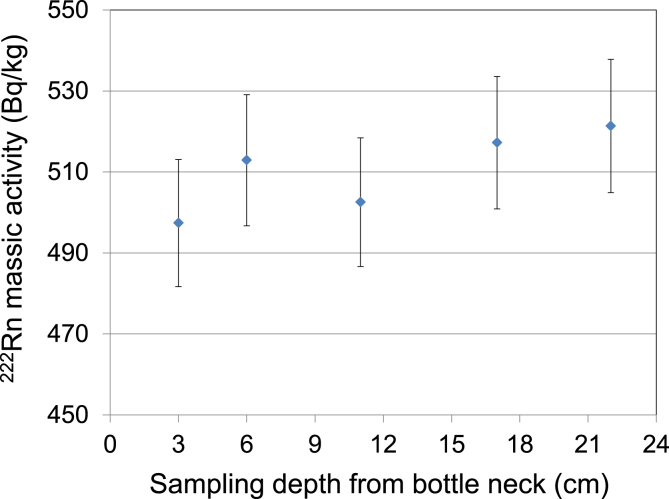
Radon in water massic activity for different sampling depths in the glass bottle measured using liquid scintillation counting. The total height of such a bottle is 22.5 cm. The combined standard uncertainty (k = 1) is given.

The radon distribution as a function of sampling depth from a sampling bottle was also investigated which is in practice to confirm the within bottle homogeneity of radon. These results are presented in Fig. 6.

It can be concluded that the results agree within their measurement uncertainties although a trend toward higher values at a greater depth can be discerned. A conservative recommendation is to collect a sub-sample from 6 cm or deeper layers. The reason for this lower value might be that after opening a bottle instant partial degassing of the sample happens which affects more the top layers closer to the ambient atmosphere as explained by Henry's law. As the partitioning coefficient indicates radon favors transfer to the air under these conditions.

This observation suggests that another possible cause for radon-loss is when test portion/sub-sample sampling is done by opening the original sampling bottle. It was also mentioned by Mazur et al. (2017) when sub-sample was transferred from an open vessel to a LSC vial.

## 4 Summary and conclusions

We found that there are numerous sources leading to radon-loss which will generate a measurement result that is too low. From a radioprotection perspective it increases the probability to put more people at risk when systematically producing too low measurement values (i.e. below the real value). Experimental studies aiming at optimizing the organization of proficiency tests for radon-in water measurements were conducted. This included pre-screening of candidate natural water samples from different water sources (well, spring, spa) to find a source that is optimal as proficiency test material. For the material characterization the most common standard measurement methods based on GS, emanometry and liquid scintillation counting were tested and verified. The methods are straightforward and capable of providing prompt measurement information about radon levels in water samples. It must be still emphasized that as ^222^Rn can easily escape the sample and measurement volume making all methods inherently less robust if care is not taken during sampling, transport and storage.

As a considerable amount of radon can be lost during sampling, this study highlighted the fact that it would be desirable to (for the first time probably) organize a PT or intercomparisons on sampling techniques at one specific site.

Since the proficiency test materials were not produced at the proficiency test organizer's site, the influence of sampling, transport and sample handling on radon-loss was tested in details and the conditions were described. The following conclusions are predominantly valid for distributing proficiency test samples but some of them should be also considered for routine laboratories/conditions to maintain sample homogeneity and stability:

(i) Radon tightness of a measurement container material has to be checked and verified especially if the sample is stored for more than one day before measurement.
(ii) The cooling and heat exposure must be also considered as it can significantly affect the measurement geometry (density and volume variation) resulting in headspace above the water sample. In case of drinking waters and natural cold spring waters we recommend that storage temperature should not be lower than sampling temperature to avoid headspace formation.
(iii) Regarding heat treatment, we did not observe significant radon-loss from glass bottles after treating them at 45 °C for three days. Despite these findings we still recommend that samples should be kept in a controlled environment until they are measured.
(iv) There is a risk of breaking glass sampling bottles if they are not heat shock resistant or their cap is made of rigid material.
(v) The influence of regular air transport on the radon massic activity was tested and no significant difference was found provided the sampling bottle was filled without headspace.
(vi) When additional sample transfer is involved (indirect measurement approach) from 1.2 up to 25% radon can be lost comparing to direct measurement results.
(vii) Radon distribution within a 22.5 cm high glass bottle was checked with LSC measurements and found to be homogeneously distributed within the measurement uncertainties.

## 5 Suggestions for best practice

On the basis of our observations we propose best practice related to sampling container material, bottle cap, storage temperature and sub-sampling for indirect analysis.

Sampling container material was proved to be crucial. It is advised to use glass as mentioned in the ISO standard (ISO 5667-3) in every case if the sample has to be stored longer than one day. There is another reason for using glass containers, namely that radon daughters tend to be absorbed on a plastic surface which causes inhomogeneous distribution in the sample and changes the measurement geometry. Furthermore, if dissolved radon concentration is relatively low and laboratory air contains relatively high concentration of radon then radon daughters can be attached to the outer wall of the container, increasing the background signals. This phenomenon is linked to the triboelectric property of materials where glass has a strong positive surface charge and plastics have usually a negative surface charge attracting positively charged particles like radon daughters. In solutions the adsorption of radon daughters depends on the surface charge-zeta potential and the pH of the water sample.

Some plastic materials like polycarbonates absorb radon and retain it in their matrix (Mitev et al., 2012). Polyethylene terephthalate (PET) as sampling bottle material was proved to be sufficient if samples are measured immediately or within a few hours after sampling.

The sampling container should be equipped with a flexible but gas tight cap to avoid accidental breaking of the glass and keep radon inside the container.

The temperature should be checked and controlled during sampling and storage. Samples should not be cooled below the sampling temperature and higher temperature exposure (>45 °C) should be also avoided, not from radon-loss point of view but to avoid breaking or causing cracks in the glass sample container.

If indirect methods are used and sample transfer is unavoidable the manipulation should be done with gas-tight syringes or tools without extra suction or vacuum. For example, immersing a pipette tip into the sampling bottle and by the principle of capillary-communicating vessels water will occupy the sub-sampling volume needed (marked on the pipette tip). Another approach could be immersing a syringe without its piston in the sampling bucket and smoothly inserting the piston underwater, the desired volume can be adjusted after taking out the syringe from the bucket.

Seeing the sensitivity of sampling for radon-in-water measurements, one can conclude that it is highly desirable to organize a PT that involves the participants travelling to the site of sampling on one given day and perform their own sampling. Discussion to organize such an event on European scale is ongoing within the JRC.
